# Factors Influencing College Students’ Generative Artificial Intelligence Usage Behavior in Mathematics Learning: A Case from China

**DOI:** 10.3390/bs15030295

**Published:** 2025-03-02

**Authors:** Wenqian Lin, Peijie Jiang

**Affiliations:** School of Mathematics and Statistics, Hunan Normal University, Changsha 410081, China; wenqianlin@hunnu.edu.cn

**Keywords:** generative artificial intelligence, mathematics learning, usage behavior, SEM

## Abstract

Generative artificial intelligence (GAI) has attracted attention in education as a tool to help college students learn mathematics. This study analyzed the factors influencing their use of GAI by applying the Unified Theory of Acceptance and Use of Technology (UTAUT) and focusing on mathematics motivation. This study involved 331 Chinese college students and used partial least squares structural equation modeling (PLS-SEM) for data analysis. The results showed that college students’ behavioral intention to use GAI to support their mathematics learning was directly influenced by performance expectancy, social influence, personal innovativeness, and mathematics motivation. Mathematics motivation, facilitating conditions, individual demand, and behavioral intention, had direct effects on college students’ use of GAI in mathematics. The most significant factor influencing both intention and behavior was mathematics motivation. Effort expectancy and individual demand did not affect the intention to use GAI in mathematics learning. In addition, there were important positive moderating effects, including individual demand, of mathematics motivation in the structural model on usage behavior and behavioral intention regarding usage behavior. The results of this study could help to identify the key influences on college students’ use of new technologies in mathematics learning and provide informative insights for the application of AI technologies in mathematics learning in the future.

## 1. Introduction

With the rapid development of technology, generative artificial intelligence (GAI), as an important type of artificial intelligence, is gradually penetrating all aspects of social life, especially in education (Yu & Guo, 2023). With its powerful language processing ability and text creativity, generative AI has unique advantages in simulating the human creative process and generating various types of content such as texts, images, audio, and videos (Țală et al., 2024), which greatly enriches methods of information generation and presentation. Currently, research on generative AI is no longer limited to breakthroughs at the technical level (Korneeva et al., 2023; Tamkin et al., 2021) or to theoretical analysis (J. Deng & Lin, 2022; Dwivedi et al., 2023), but is paying more attention to exploring its potential and value in practical applications of education, which is becoming the focus of attention around the world.

In mathematics education, the integration of GAI has its certainty and importance. As the theoretical foundation of artificial intelligence, mathematics has the logic and abstraction to provide a rich application scenario for GAI (Genesereth & Nilsson, 2012). At the same time, when traditional mathematical teaching modes are facing the problems of limited resources, single teaching styles, and insufficient situational activities (Adler, 2021; Lessani et al., 2017), GAI provides a new solution for mathematical teaching with its improved teaching methods and strategies (Topsakal & Topsakal, 2022), powerful integration of curriculum resources, and intelligent tutoring capabilities (Qadir, 2023). By intelligently analyzing students’ learning data and habits, GAI can provide students with personalized learning content, achieve accurate tutoring, and effectively enhance students’ learning efficiency and interests (Ruiz-Rojas et al., 2023). As a new technology, the integration of GAI into students’ mathematical learning process is an inevitable trend in the future of mathematics education. Therefore, understanding students’ willingness to accept and use GAI technology in mathematics learning has become an important issue to be explored. It can help to analyze the many factors influencing students’ use of GAI-supported mathematics learning. It is of great value in optimizing the allocation of teaching resources and scientifically applying GAI technology to achieve personalized learning and tutoring for students (A. Y. Huang et al., 2023).

In education, motivation, as a dynamic factor, has an important influence on students’ willingness to learn and behavior regarding learning, from the point of view of the internal psychological process of learning activities (Chan et al., 2015). According to psychologists, motivation can stimulate, maintain, regulate, and guide people to engage in a certain activity (Wolters, 2003), and it is the driving force for individuals to complete one thing successfully. However, students’ motivation in mathematics is different from general academic motivation due to the discipline-specific nature of the subject (Graham & Weiner, 2012). As a result, it is necessary to investigate the influence of mathematics motivation on students’ use of GAI to support their mathematical learning. The current studies have also indicated that there is a very limited amount of research exploring learner acceptance by integrating motivation into technology acceptance models (Nikou & Economides, 2017), and even more notably, investigations on the moderating role of motivation in the models are critically lacking, which is a gap in the existing literature.

Considering that college students generally have a higher independent learning ability and adaptability to new technologies than primary and secondary school students, they are willing to try new learning tools and apply them to mathematics learning. Therefore, this study selected college students enrolled in higher education as the research object and adopted the Unified Theory of Acceptance and Use of Technology (UTAUT) as the theoretical basis to examine the following research questions:

(1) What factors influence college students’ intention to use and behavior regarding the use of GAI to support their mathematics learning?
(2) Does mathematics motivation influence college students’ intention to use and behavior regarding the use of GAI to support their mathematics learning?
(3) Does mathematics motivation moderate the relationships between the pathways in the model of college students’ use of GAI?

## 2. Literature Review and Hypothesis Development

### 2.1. Generative Artificial Intelligence in Mathematics Education

With the release and improvement of ChatGPT, GAI technology has gained worldwide attention and popularity for its powerful data analysis capabilities and conversational interaction. It has profoundly impacted the interrelationships between technology, pedagogy, and content (Ning et al., 2024), in particular providing opportunities for democratizing the distribution of educational resources and personalized learning (Kumar, 2024), which has significantly influenced students’ digital mathematics learning.

Taani and Alabidi (2024) investigated mathematics teachers’ use and perceptions of AI applications to enhance students’ learning experience in mathematics classrooms using ChatGPT. They found that GAI technologies can be applied in various ways, such as generating mathematical examples, assessing the difficulty of test questions, providing explanations of proofs, and supporting problem-solving. By analyzing the online educational system MathE platform, Azevedo et al. (2024) demonstrated the value of next-generation AI technologies for understanding students’ mathematics learning patterns, gaining a global perspective on mathematics education, and facilitating the development of mathematics learning tools. Luo et al. (2024) explored the value of generative teacher rubrics in improving students’ self-regulated learning abilities and motivation in a mathematics classroom based on a generative AI platform and intelligent diagnostic data. Barana et al. (2023) conducted a study on the effectiveness of assessment in solving tasks with AI tools, exploring six combinatorial problems using ChatGPT, and the results of the study showed that the integration of AI technology in learning and assessment helped to solve mathematical problems and develop critical thinking. Lo (2023) pointed out that GAI has potential for generating course materials and acting as a virtual tutor for students. Still, its performance in the subject area of mathematics is deficient compared to programming and economics. It can be seen that the use of GAI technology in mathematics learning not only helps to build personalized adaptive teaching environments for intelligent education, but also helps students to improve their mathematical understanding, promotes the in-depth learning of mathematical knowledge, and provides a new approach to carry out accurate and continuous learning assessments in teaching. Due to the pioneering application of emerging technologies in teaching in higher education institutions, college students have become the main group currently making practical use of GAI. Therefore, it is necessary to understand the basic situation of college students using generative artificial intelligence to support mathematics learning and explore the many factors that affect college students’ adoption of GAI in mathematics courses to provide a valuable reference for higher education and students at other educational levels to effectively use generative artificial intelligence technology in mathematics learning.

### 2.2. Characteristics of College Students’ Mathematics Learning

In the current information-based educational environment, college students are experiencing significant changes in how they learn mathematics. They are no longer limited to the traditional passive acceptance of the classroom mode, but actively use a variety of channels, such as network resources, online courses, academic forums, etc., for independent learning (Han, 2022). Diversified learning methods broaden the access to mathematical resources for college students and effectively promote the development of personalized and adaptive learning.

With the continuous development of science and technology, the application of technology has long been an important feature of college students’ mathematical learning. The emergence of generative AI technology makes this feature even more obvious. First, a comparative study (Gurevich & Barchilon Ben-Av, 2023) found that integrating technology into college students’ mathematics learning can improve the quality of learning. Another empirical study investigating how information and communication technology (ICT) can be integrated into remedial mathematics teaching and learning confirmed this view (Chen & Wu, 2020). Second, university mathematics is a subject that combines theory, computation, and application, which are difficult to learn and require significantly higher order knowledge. Computer graphics and simulation technology can visualize these to reduce the difficulty of acquiring knowledge. Third, college students studying higher mathematics are often asked to answer or prove a complex problem from many angles. GAI is an effective way to improve their mathematical understanding and creativity (Stupel & Ben-Chaim, 2017). Generative AI technologies can quickly provide multiple solutions and methods using complex algorithms and models, helping students to develop creative thinking. Finally, if many previous technologies have raised the threshold for their use by college students in mathematics learning because of the complexity of their access and operation, generative AI has better avoided this problem. The simple and easy-to-use characteristics of GAI based on a large language model make it universally available, and it can support college students’ learning regardless of the limitations of time and space. In addition, compared to basic education, AI is more widely used in college classrooms (F. Li & Wang, 2023). College students have a relatively high independent learning ability, and technological advantages are important in promoting their in-depth mathematics learning. In summary, for college students as a group with a strong acceptance of new technologies and tools, using technology to support mathematics learning has become an inevitable trend. Studying the influencing factors of college students’ use of generative artificial intelligence to support mathematics learning is of great practical significance and theoretical value.

### 2.3. Unified Theory of Acceptance and Use of Technology

In 2003, Venkatesh et al. proposed the Unified Theory of Acceptance and Use of Technology (UTAUT) based on eight theoretical models (Venkatesh et al., 2003). UTAUT consists of four core constructs influencing users’ acceptance and use of technology: performance expectancy, effort expectancy, social influence, and facilitating conditions. UTAUT has been used in a wide range of settings, from the development of the Internet (Zhou et al., 2010) to the widespread adoption of educational technologies such as tablet teaching and e-textbooks (Cacciamani et al., 2018) to new explorations in the age of artificial intelligence (Du & Lv, 2024). At different stages of development, UTAUT models have continuously adapted to new technological environments to explain and predict users’ technology acceptance and usage behavior. AI technology has significantly improved student learning outcomes in mathematics (Patel et al., 2023). However, current research mainly focuses on the output side of GAI, including how to improve GAI algorithms and integrate GAI into instructional resources to improve teaching efficiency. Fewer studies have examined student acceptance of GAI from the receiving side, and the factors influencing students’ use of GAI-assisted learning. Therefore, this study addresses this research gap with further research based on UTAUT. Considering that individuals’ differentiated characteristics may influence students’ overall usage behavior, this study adds the two variables of personal innovativeness and individual demand to the basic UTAUT model to form an extended UTAUT model with eight constructs.

#### 2.3.1. Performance Expectancy (PE)

Performance expectancy is the degree to which users believe that using the technology will help them be more efficient and effective (Venkatesh et al., 2012). Performance expectancy is one of the key factors influencing users’ acceptance and use of technology. One study emphasized that performance expectancy significantly and positively affects college students’ intention to accept AI-assisted learning environments (Wu et al., 2022). This impact is even more evident in the ability of technological devices to solve difficulties and provide effective ideas for students to learn their subjects (Wijaya et al., 2022c). In this study, performance expectancy refers to the extent to which college students believe that AI will help them to improve the efficiency and effectiveness of their mathematics learning. Therefore, the following hypothesis is proposed in this study:

**H1:**  *Performance expectancy directly affects college students’ behavioral intention to use GAI to support their mathematics learning.*

#### 2.3.2. Effort Expectancy (EE)

Effort expectancy is defined as the degree to which the system is perceived as easy to use by the user, including the ease of learning to use, operate, and maintain the technology (Venkatesh et al., 2003). Previous research has shown that effort expectancy is the strongest predictor of students’ behavioral intention to use mobile learning (Abu-Al-Aish & Love, 2013). For this study, effort expectancy refers to the extent to which students perceive the GAI platform to be easy to use. If students perceive that using GAI requires more effort, their intention to use it may decrease. Conversely, students’ intention to use this technology increases when they are given an interactive experience to demonstrate the ease of use (Zeng, 2019). Therefore, the following hypothesis is proposed in this study:

**H2:**  *Effort expectancy directly affects college students’ behavioral intention to use GAI to support mathematics learning.*

#### 2.3.3. Social Influence (SI)

Social influence refers to how individuals perceive that people in their significant social relationships will expect them to use a new technology (Venkatesh et al., 2003, 2012). Social influence is categorized into two dimensions: superior and peer influence. Empirical studies have shown that students’ willingness to use e-library services depends on social influence (Awwad & Al-Majali, 2015). This study combines the key dimensions of social influence to examine its impact on university students’ use of GAI to support their mathematics learning, and proposes the following hypothesis:

**H3:**  *Social influence directly affects college students’ behavioral intention to use GAI to support mathematics learning.*

#### 2.3.4. Facilitating Conditions (FC)

Facilitating conditions are the extent to which an individual believes that an organization or related technological infrastructure is available to support their use of technology (Venkatesh et al., 2003, 2012). A study of Indonesian university students using ChatGPT for learning found that facilitating conditions were the strongest predictor of university students’ use of generative AI technology (Habibi et al., 2023). In contrast, a study of students using GAI for foreign language learning found that facilitating conditions were not significantly related to students’ behavioral intention to use GAI (Zheng et al., 2024). To investigate whether facilitating conditions have an impact on college students’ technology usage behavior in mathematics learning, the following hypothesis is made:

**H4:**  *Facilitating conditions directly affect college students’ behavioral intention to use GAI to support mathematics learning.*

#### 2.3.5. Personal Innovativeness (PI)

This concept was introduced by Agarwal and Karahanna (2000) in the context of information technology to reveal the state of deep individual engagement with software, and they stated that personal innovativeness is an important determinant of cognitive absorption. It has been found that personal innovativeness is the willingness of users to try new technologies, and that individuals with higher levels of innovativeness are more willing to adopt new tools compared to individuals with lower levels (Lu et al., 2005; Wijaya et al., 2022b). In an educational context, personal innovativeness is a necessary complementary construct in the UTAUT model that significantly influences technology adoption (Tian et al., 2024). It has been supported by numerous studies (e.g., Sidik & Syafar, 2020; Twum et al., 2022; Zhu & Huang, 2023). In this study, students with high personal innovativeness are expected to be more willing to experiment and more positive in their willingness to use GAI in their mathematics studies. Therefore, the following hypothesis is proposed in this study:

**H5:**  *Personal innovativeness directly affects college students’ behavioral intention to use GAI to support their mathematics learning.*

#### 2.3.6. Individual Demand (ID)

Some studies have defined individual demand as a group’s need for personalized information display and the expression of personal characteristics when using a particular technology (B. Li, 2020). Personalized content can directly influence users’ willingness to use and actual behavior regarding the use of a technology (S. Deng et al., 2019). The synergy between AI and education is expected to lead to personalization in education, with GAI tailoring the educational experience to each student’s unique needs, preferences, and pace (Ayeni et al., 2024). A recent study (Gao, 2023) has shown that GAIs can personalize learning recommendations for students based on their success in meeting their specific learning needs, study habits, and learning abilities. Thus, it can be concluded that the availability of personalized learning requirements impacts both students’ willingness to use and behavior regarding the use of GAI. Therefore, the following hypotheses are proposed in this study:

**H6:**  *Individual demand directly affects college students’ behavioral intention to use GAI to support mathematics learning.*

**H7:**  *Individual demand directly affects college students’ usage behavior in applying GAI to support mathematics learning.*

#### 2.3.7. Behavioral Intention (BI) and Usage Behavior (UB)

Behavioral intention is an individual’s subjective decision to perform a task, which reflects the individual’s willingness to adopt a particular behavior and directly influences the individual’s usage behavior (Venkatesh et al., 2003). In this study, behavioral intention is defined as students’ intention to use GAI for mathematics learning. It has been shown that the stronger an individual’s intention to use an advanced technology to help them learn, the more likely they are to use that technology multiple times while learning a subject (Alzahrani et al., 2019; Wijaya et al., 2022c; Yuan et al., 2023). Therefore, the following hypothesis is proposed in this study:

**H8:**  *Behavioral intention directly affects college students’ usage behavior in applying GAI to support mathematics learning.*

### 2.4. Mathematics Motivation (MM) to Apply GAI for Learning

#### 2.4.1. Theoretical Analysis

Motivation, as the internal drive of learning activities (Deci et al., 1991), is an important condition for initiating, maintaining, and completing students’ learning behaviors and closely affects students’ learning attitudes and achievements. In mathematics learning, students’ motivation is also related to the teaching and learning of mathematics. Some empirical studies (Gottfried et al., 2007; Hu et al., 2021) have shown that mathematics motivation has a significant impact on the improvement of students’ academic performance. Motivation, as a dynamic factor, plays a role in whether a learner adopts a particular technology for learning and what kind of meaningful learning behavior occurs with the help of technology (Kazu & Kuvvetli, 2023). Therefore, it is necessary to examine the influence of mathematics motivation as a key variable in college students’ GAI-assisted mathematics learning.

Self-determination theory (SDT), one of the most influential theories in motivational psychology, is a theory of the motivational processes in human self-determined behavior proposed by Deci and Ryan (1985). Based on recognizing human organic needs, SDT emphasizes the degree of self-determination of human behavior and distinguishes the types of motivation for self-determined behavior. The theory divides motivation into intrinsic, extrinsic, and amotivation (Vallerand & Blssonnette, 1992). Intrinsic motivation is an innate human tendency to seek novelty and challenge, to develop and exercise one’s abilities, and to explore and learn. It is closely related to the individual’s internal factors, such as interest and satisfaction. Extrinsic motivation is the tendency to engage in an activity to achieve a separable outcome, such as obtaining a high score or avoiding punishment. For Chinese parents and students, pursuing further education is undoubtedly one of the most significant external motivations (Liu & Lin, 2010). Amotivation represents the low end of SDT motivation, characterized by a lack of intention to act and the absence of any external or internal regulatory behaviors to ensure that the activity is performed properly. Motivation theories such as SDT view motivation as a continuous spectrum or dimension. However, amotivation, as a state of motivational absence in this spectrum, is often not included as a motivational type in academic research (Ryan & Deci, 2020). Therefore, based on SDT, this study categorizes mathematical motivation into intrinsic and extrinsic.

#### 2.4.2. Proposed Model

Based on the social cognitive modeling theory, Pintrich et al. developed the motivated strategies for learning questionnaire (MSLQ), primarily designed for college student populations (Pintrich, 1991; Pintrich et al., 1993). The MSLQ articulates and argues that the strength of an individual’s motivation triggers the execution of good or bad learning strategies, directly affecting learning behavior. The instrument is modular and consists of two parts, motivation and learning strategies, with 15 subscales designed to assess the motivational orientation of university students and their use of different learning strategies in courses. Thousands of students have tested the validity of the MSLQ, which has garnered more than ten million citations in academic works. It has been widely used to study college students from different countries, and a conclusive meta-study also confirms that the MSLQ is a reasonably reliable measurement tool (Credé & Phillips, 2011). Pintrich (1991) states in the manual that the 15 subscales of the instrument can be used together or separately. In the present study, two relevant subscales from the motivation section of the MSLQ were used: the intrinsic goal orientation (IGO) scale to measure the intrinsic motivation of college students and the extrinsic goal orientation (EGO) scale to measure the extrinsic motivation.

**Figure 1:**
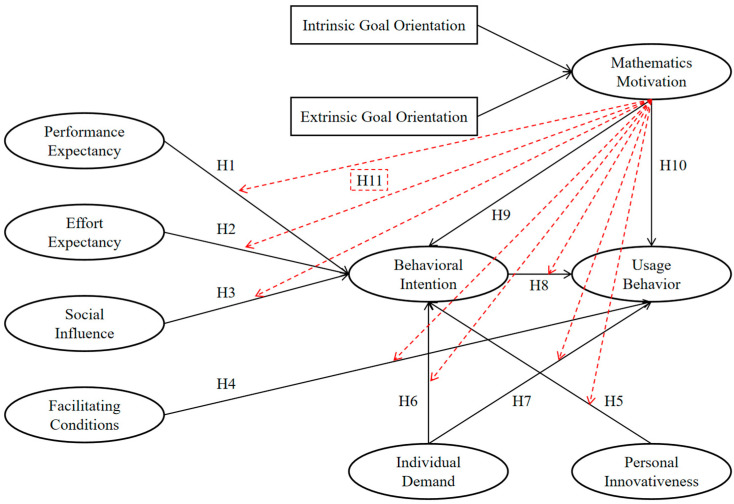
The proposed conceptual model and hypotheses. Note: The red dotted line indicates the moderating effect.

Therefore, this study adds mathematics motivation as an additional important variable in the extended UTAUT model to explore the direct effect of mathematics motivation on college students’ behavioral intention to learn mathematics using GAI and their usage behaviors. In addition, according to the results of existing studies (Hsu, 2023; Zheng et al., 2024), motivation can enhance or reduce the effects of factors such as performance expectation and facilitation on technology acceptance and actual use. This suggests that mathematics motivation is also an important moderating variable in the research model of willingness to adopt and behavior regarding the use of new technologies. Consequently, the study proposes the following hypotheses (Figure 1):

**H9:**  *Mathematics motivation directly affects the behavioral intention of college students to use GAI to support their mathematics learning.*

**H10:**  *Mathematics motivation directly affects college students’ usage behavior in applying GAI to support their mathematics learning.*

**H11:**  *Mathematics motivation moderates all path relationships in the model of college students’ use of GAI to support mathematics learning.*

## 3. Methodology

This study employs quantitative analysis to scrutinize the level of acceptance and the factors influencing college students’ utilization of GAI in supporting their mathematics learning by testing the initial hypotheses through the application of structural equation modeling (SEM) framework. Many existing studies have confirmed that the SEM method is suitable for analyzing and explaining the relationship between variables and testing the fit of models and is an effective test for determining the intention to adopt a technology (Dong et al., 2023; Tondeur et al., 2020).

### 3.1. Study Instrument

The original English questionnaire used in the study was translated by two professors and synthesized and validated by three graduate students and two experts in English studies. The questionnaire consisted of three parts. The first part was used to find out the basic information of all participants, including gender, grade, major, knowledge of GAI, and whether they had used GAI in mathematics learning. The second part was designed to investigate the acceptance and use behavior of GAI among college students in math learning, and which was filled out by the respondents who had used GAI. The section was designed around the eight constructs of the extended UTAUT model with 25 question items (Table A1). The third section focused on students’ motivational orientations toward using GAI to support mathematics learning and was also completed by students who had experience using GAI. Under the guidance of the MSLQ manual, this study adapted the original scale to consider the characteristics of the mathematics program and the general mathematics learning habits of college students in China. The final result was ten items measuring mathematical motivational orientation during GAI use, which consisted of five items for intrinsic and five items for extrinsic goal orientation used together (Table A2). The second and third parts were rated on a 5-point Likert scale, with 1–5 indicating strongly disagree, disagree, fair, somewhat agree, and strongly agree, respectively.

In this study, a full pilot pre-survey was conducted in early August 2024 before conducting the formal survey. Based on the results of the pre-survey, changes were made to the content of the conceptualization, with a total of five items deleted, four items added, and some question wording modified to make them clearer and more explicit. The reliability and validity of the questionnaire were then comprehensively evaluated by four experts and four researchers, and the final version, which could be used in the formal survey, was developed after improvement.

### 3.2. Data Collection

In this study, whole cluster random sampling was used, and the selected respondents were college students enrolled in higher education institutions who were required to take mathematics courses, including undergraduate students, master’s students, and doctoral students. They were mainly from Hunan Province in the central region of China and Zhejiang Province in the eastern region. This is because in China, compared with the western region, the central and eastern regions have high-quality educational resources, an open policy environment for the development of higher education, and stable economic development (X. X. Li & Chen, 2023). Hunan Province and Zhejiang Province, as representative provinces of the central and eastern regions of China, respectively, have a relatively high quality of mathematics education in higher education institutions and actively promote the digitalization of education, focusing on developing students’ mathematical innovation ability and information literacy.

The survey started on 15 September 2024 and lasted for three weeks, with 477 participants’ data collected (Table 1). Of the total participants, 366 (76.73%) indicated that they had used GAI in mathematics learning, which indicated that GAI had a high prevalence in the college student population and initially reflected that most college students were willing to accept GAI and try to use technology to support their mathematics learning. When screening the valid values for data analysis, after removing the data from 111 participants who did not use GAI, 35 incomplete values were also excluded, and a total of 331 participants’ data were obtained for analysis.

Of the total participants, 121 were male and 210 were female. The participants were evenly distributed across all levels of study, from undergraduate to graduate. Among them, 66.47% were from a mathematics major, 20.54% were from a business major, and there were also some from engineering and electronics majors. Regarding the frequency of use, half of the participants used GAI occasionally (53.17%) in their mathematics learning, and 26.59% used it frequently. Table 1 shows the complete demographics.

### 3.3. Data Analysis

Based on the partial least squares structural equation modeling (PLS-SEM) approach, this study used the SPSS 27 and SmartPLS 3.2.9 tools to analyze the data and to test the significance of the coefficients of the hypothesized paths in the model to develop measurement models that could systematically assess the factors that influence college students’ use of GAI in their mathematics learning. In contrast to covariance-based structural equation modeling (CB-SEM), PLS-SEM can test the fit of research models, explain the relationships among variables, and handle complex structural models with multiple constructs (Dash & Paul, 2021). In addition to theory validation, Hair et al. pointed out that PLS-SEM is also suitable for model exploration and development (Hair et al., 2019).

First, it should be understood that SEM analysis required that the data be tested for normality (Ali et al., 2018), and SPSS was used in this session. Second, the analysis of PLS-SEM included measurement model and structural model tests. On the one hand, the measurement model test mainly evaluated the structural model’s reliability, convergent validity, and discriminant validity. On the other hand, the structural model test explained the model fit, explanatory power, path coefficients, and significance.

### 3.4. Ethical Considerations

The study questionnaire was designed to be completed anonymously. At the beginning of the questionnaire, respondents were informed of their right to choose whether or not to participate in the survey, and all participants followed a highly voluntary principle to participate in the survey. In addition, the questionnaire instructions informed participants of the purpose of the survey and the content and use of the data, and the survey was conducted under the principle of informed consent. The research team ensured that the information and responses would be kept confidential and not disclosed to third parties.

## 4. Results

The data analysis and results are divided into three sections. Firstly, the results of the descriptive statistics are presented to analyze the normality of the data. Secondly, the measurement model is evaluated. Finally, the structural model is analyzed, and hypothesis testing is performed.

### 4.1. Normality Analysis

Table 2 shows the descriptive statistics, including the mean, standard deviation, skewness, and kurtosis. According to the results, it can be found that the skewness and kurtosis values range from −0.532 to 0.039 and −0.528 to 0.091, respectively, which are in line with the threshold ranges of ±3 and ±10, and both are close to 0 (Ho & Yu, 2015; Wijaya & Weinhandl, 2022). It is indicated that the data follow a normal distribution and can be analyzed using structural equation modeling.

### 4.2. Measurement Model

The analysis of a measurement model requires an assessment of the reliability and validity of the model. Reliability represents the degree of internal consistency and reliability of the measurement questionnaire, which is mainly measured by Cronbach’s Alpha. In general, the acceptable value of Cronbach’s Alpha is 0.7. If it exceeds 0.8 but does not exceed 0.95, the measurement instrument has high reliability (Henseler et al., 2015). In this report, Cronbach’s Alpha for the constructs ranged from 0.813 to 0.930 (Table 3), indicating a high level of reliability for each latent variable in the model.

The report also examined the three measures of factor loading, composite reliability (CR), and average variance extraction (AVE) to assess convergent validity. The results showed that the factor loadings for all questions were greater than 0.7, and the AVE values for each construct ranged from 0.613 to 0.842, which exceeded the acceptable criterion of 0.5. According to the strict criterion, the CR value must be greater than 0.7, and all constructs in the report met this condition. It is indicated that the model has good convergent validity (Hair et al., 2021). In summary, the instrument can be judged to have satisfactory internal consistency and stability.

### 4.3. Confirmatory Factor Analysis

Discriminant validity is another important validity indicator in structural equation modeling. While convergent validity reflects the internal correlation between different items, discriminant validity requires that the different items also maintain relative independence and can effectively discriminate between different traits or concepts. According to the PLS-SEM, the Fornell–Larcker criterion was uesd in this study to assess the discriminant validity of the latent variables (Henseler et al., 2015). Table 4 shows the correlation coefficients between the latent variables, where the position of the matrix diagonal is the square root of the AVE. According to the Fornell–Larcker criterion, if each AVE square root value in the table is greater than the absolute value of the other correlation coefficients, then it can be assumed that there is good discriminant validity between each latent variable and the other latent variables.

However, it is not sufficient to use the Fornell–Larcker criterion as the only assessment of the discriminant validity of structural equation modeling; the Heterotrait–Monotrait Ratio (HTMT) should also be examined (Henseler et al., 2015). This is because HTMT values are intended to further enhance the accuracy of validity assessment by assessing the heterogeneity and monotrait between constructs. According to the requirements, the HTMT value should not exceed 0.9. The HTMT statistics in Table 5 indicate that the discriminant validity of the model meets the criterion.

Discriminant validity is an important aspect of confirmatory factor analysis, while another aspect is the assessment of model fit (Hair et al., 2012). In PLS-SEM, model fit is commonly measured by the standardized root mean square residual (SRMR) and normed fit index (NFI). Typically, the acceptable criterion for the NFI is greater than 0.8. An SRMR less than 0.05 is considered a good fit, while less than 0.08 is considered an acceptable fit criterion (Dash & Paul, 2021). The analysis results show that the model has an SRMR value of 0.054 and an NFI value of 0.813, which are within the acceptable criteria. Although SRMR is a widely accepted indicator of model fit and NFI is also used in structural equation modeling, the goodness of fit (GOF) is often used to measure global fit because PLS-SEM is more predictive-oriented. GOF values of 0.1–0.25, 0.26–0.36, and above 0.36 indicate the model has weak, moderate, and good global fit, respectively (Wetzels et al., 2009). The GOF metric is related to the mean variance extracted and the R^2^ value, and is calculated as GOF = $\sqrt{\bar{\mathrm{Communality}}•\bar{R^{2}}}$ (Moradi et al., 2018). According to the output data, the GOF value is 0.619. Therefore, it can be comprehensively judged that the research model has an excellent fit, which can better reflect the data characteristics and provide good prediction results.

### 4.4. Structural Model and Hypothesis Test

Table 6 shows the path coefficients, sample means, standard deviations, t-values, and *p*-values representing the level of significance corresponding to the initial hypotheses of the model. Based on the evaluation criteria of 1.96 < | t | < 2.58 and *p* < 0.05 (C. H. Huang, 2021), eight hypotheses were supported, and two hypotheses were rejected.

The results showed that performance expectancy and social influence had a significantly positive effect on college students’ behavioral intention to use GAI to support their mathematics learning (β = 0.178, t = 2.813, *p* = 0.005; β = 0.120, t = 2.042, *p* = 0.041), and H1 and H3 were supported. Personal innovativeness also significantly influenced students’ behavioral intention to use GAI for mathematics learning (β = 0.257, t = 3.815, *p* = 0.000), supporting H5. Individual demand and facilitating conditions positively influenced the usage behavior of college students in adopting GAI technology for mathematics learning (β = 0.232, t = 3.703, *p* = 0.000; β = 0.290, t = 5.117, *p* = 0.000), which provides support for H7 and H4. Mathematics motivation had a strong positive correlation with both the behavioral intention and usage behavior of university students regarding learning mathematics with the help of GAI (β = 0.406, t = 4.672, *p* = 0.000; β = 0.255, t = 3.276, *p* = 0.001), which supports H9 and H10. Both effort expectancy and individual demand were not significantly correlated with behavioral intention (β = 0.046, t = 0.567, *p* = 0.571; β = −0.067, t = 0.873, *p* = 0.383), and H2 and H6 were not valid. This indicates that effort expectancy and individual demand do not affect college students’ intention to use generative AI to support mathematics learning. Also, as expected, college students’ behavioral intention to use GAI in math learning directly affected their usage behavior, and H8 holds (β = 0.204, t = 2.527, *p* = 0.012).

**Figure 2:**
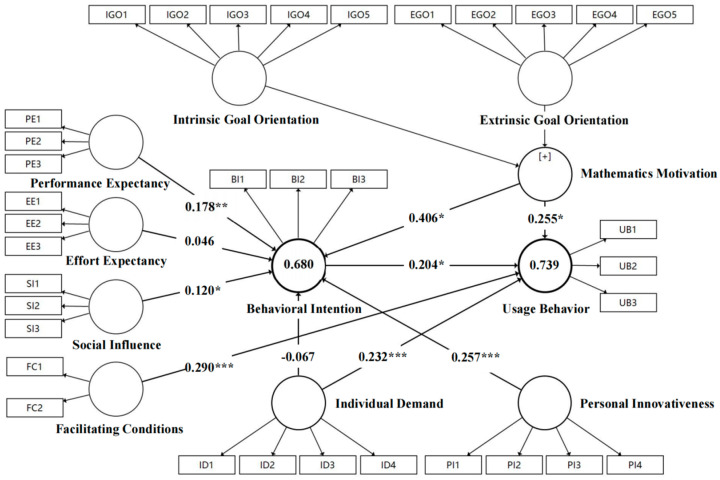
The final structural model with R^2^, path coefficients, and *p*-values. Note: * *p* < 0.05, ** *p* < 0.01, *** *p* < 0.001.

The structural model also provided a variance explanation (R^2^) of the relationship between the dependent and independent variables, which reflected the extent to which the independent variables could explain the model’s dependent variable. R^2^ values greater than 0.67, 0.33, and 0.19 indicate that the model has a high, moderate, and weak level of explanation, respectively (Khan et al., 2022). As shown in Figure 2, the proposed model could explain 68% and 73.9% of the variance in college students’ behavioral intention and usage behavior regarding learning mathematics using GAI, respectively. This indicates that the model had excellent explanatory power and almost 70% agreement with the UTAUT model (Venkatesh et al., 2003).

### 4.5. Direct, Indirect, and Total Effect

Information on the direct, indirect, and total effects between the constructs can be found in Table 7. The total effects on behavioral intention are all caused by direct effects. The variable with the largest direct effect on behavioral intention was mathematics motivation, with a total effect value of 0.406. The next variable with a large effect was personal innovativeness, with an effect value of 0.257. Social influence and performance expectancy also directly affected behavioral intention, but the effect was relatively small. Usage behavior was both directly and indirectly affected by the variables. Mathematics motivation, facilitating conditions, individual demand, and behavioral intention all had large direct effects on usage behavior, with direct effect values of 0.338, 0.290, 0.218, and 0.204, respectively. The other determinants all indirectly affected usage behavior, but the effect sizes were weak, with all less than 0.1 (Wijaya et al., 2022a). It can be seen that mathematics motivation also had the largest effect size on usage behavior.

### 4.6. Moderating Effect

After centering the variables and constructing regression equations with interaction terms, this study used linear regression for moderating effects analysis (Sun et al., 2023) to test each possible moderating effect in H11. Only two of the eight moderating hypotheses generated by mathematics motivation are supported by testing the significance of the model’s interaction terms. The results of the existing moderating effects are reported in Table 8. Among them, mathematics motivation has a positive moderating effect on “H7: Individual Demand→Usage Behavior” (β = 0.079, *p* = 0.022). This means that mathematics motivation significantly affects the strength of the relationship between individual demand and usage behavior. When college students have strong mathematics motivation, they seek learning resources and ways to meet their personalized needs and thus actively use various learning tools. Then, there is also a significant positive moderating effect of mathematics motivation on “H8: Behavioral Intention→Usage Behavior” (β = 0.022, *p* = 0.015). This suggests that mathematics motivation, as an intrinsic driver, can significantly influence an individual’s intention to use mathematical tools and further modulate that intention into the actual process of performing the behavior.

## 5. Discussion

Generative artificial intelligence (GAI) has shown great potential and value in education, especially in mathematics. The use of GAI for mathematics learning is very helpful for many of the contents of college mathematics courses. Although the GAI technology represented by ChatGPT has attracted widespread attention, and some scholars have begun to study the instructional application of GAI (Jeon & Lee, 2023; Peres et al., 2023), few studies currently investigate and analyze the influencing factors of college students’ use of GAI to support their mathematics learning. This study adds the variables of personal innovativeness and individual demand to the original UTAUT model, additionally considers the intrinsic influence of mathematics motivation on college students’ mathematics learning, and uses the PLS-SEM method to determine the intention and behavioral factors that influence Chinese college students’ use of GAI for mathematics learning. The results of the overall model evaluation indicate that the structural model proposed in this study explains and validates the factors influencing college students’ intention to use and behavior regarding the use of GAI in mathematics learning to an extent of about 70%.

Performance expectancy (PE) and social influence (SI) positively affect college students’ intention to use GAI in mathematics learning. This is consistent with the findings of previous studies (Hoque & Sorwar, 2017; Khlaif et al., 2024; Tam et al., 2020; Zheng et al., 2024) that these two variables influence the formation of behavioral intention. It is found that students’ expectations of the learning tools influence their intention to use them. When college students perceive that GAI will positively contribute to their mathematics learning, they are more willing to use the tool and show a higher satisfaction with it. Although self-perception is an important reason for students to use GAI autonomously, the influence of external factors should not be ignored. As active participants in technology-enhanced learning environments, students’ perceptions of digital tools are inherently shaped by the socio-technical context of their academic experiences (Coman et al., 2020). This necessitates a systematic integration of instructor guidance and the critical evaluation of media discourse to optimize technology adoption in educational practice.

Second, personal innovativeness (PI) also impacts students’ use of GAI tools in mathematics learning. This was confirmed in Wijaya’s empirical study (2022b), which used the UTAUT model to predict the factors influencing pre-service teachers’ behavioral intention to implement STEM education. This suggests that innovative individuals are curious about new things (Boaler, 2016) and are willing to try new techniques in mathematics learning (Trust, 2017). Highly innovative students also tend to continue to explore the various uses of GAI in their mathematics activities, actively seeking out GAI tools, and integrating them into the learning process. It is easy to see that students with innovative literacies are more likely to develop the behavior of using GAI in mathematics learning. In turn, using GAI supports the engagement of the creative processes necessary for their mathematics learning. Educators should focus on developing students’ creative skills in their daily mathematics programs and stimulate their creativity in mathematics learning through technology. It is an effective way to support the development of their mathematics skills.

Among the four factors influencing college students’ usage behavior in applying GAI for math learning, mathematics motivation (MM) has the greatest positive influence. Secondly, facilitating conditions (FC) are the second most important factor influencing the actual use of GAI. This is supported by many studies (e.g., Bervell & Arkorful, 2020; Rusman et al., 2024; Shirolkar & Kadam, 2023). However, in some studies, facilitating conditions did not significantly affect the usage behavior of a group of teachers in terms of using a particular technology (Abd Rahman et al., 2021; Alshammari, 2021; Gellerstedt et al., 2018). The possible reason for this is that the teacher population already has a certain level of technological proficiency and experience in using instructional technology, and the convenience of using technology does not play a key role in using that tool. Even if the convenience of the technology is low, teachers may overcome the difficulties and continue to use the technology because of their instructional demands. However, college students tend to be more concerned with the convenience and efficiency of the math learning tool, and a lack of convenience may lead them to abandon the use of the technology in question because of the difficulties they encounter in using it. These differences suggest that the characteristics and needs of different groups of users need to be fully considered when choosing a technology for mathematics tasks (Radović et al., 2019).

On the other hand, individual demand (ID) also significantly and positively influences the behavior of college students in using GAI for mathematics learning. Individual learning needs, such as resource availability and subjective interest, influence whether college students adopt a particular technology in mathematics learning. Digital technologies are transforming teaching, learning, and assessment in mathematics education (Engelbrecht & Borba, 2024), providing new opportunities for personalized learning where learners can benefit from adaptive systems (Weigand et al., 2024). For college students with varying levels of academic achievement in mathematics, GAI provides personalized learning support by gradually understanding learners’ learning needs through an ongoing conversational process. Such individualized demands also often influence the persistence of students’ GAI-using behaviors in mathematics activities.

Although individual demand (ID) significantly influences college students’ behavior of using GAI, it does not directly influence college students’ behavioral intention to use GAI in mathematics learning. This suggests that intrinsic needs strongly influence college students’ operational behaviors regarding mathematics learning, and when they have an intrinsic personalized need to use GAI in mathematics learning, this will, to a large extent, directly generate usage behaviors.

Consistent with the expected results, the behavioral intention (BI) to use GAI directly influences usage behavior (UB). Contrary to expectations, effort expectancy (EE) does not influence the generation of behavioral intention to use GAI by college students in mathematics learning. The reason for this may be that GAI is not difficult for college students to operate, and they do not need to expend much effort to learn how to use it, thus reducing the effect of effort expectations on their behavioral intention to use GAI for learning. This is also supported by existing research findings (Azhar et al., 2021; Holzmann et al., 2020).

A surprising finding, however, is that mathematics motivation (MM) consistently produces the largest positive effect on both students’ behavioral intention to use GAI to support their mathematics learning and their usage behavior. Mathematics motivation, from an intrinsic value orientation, suggests (Chiu & Wang, 2008; Raman et al., 2022) that individuals are more likely to use GAI for in-depth mathematical exploration when they are interested in the mathematical materials provided by GAI or when they gain a sense of accomplishment in their mathematical learning through the assistance of GAI. In terms of extrinsic goal-directed mathematics motivation, if college students improve their academic performance in mathematics by using a new learning tool or gain recognition from their teachers and peers as a result of using the tool in a mathematics project, they will also be motivated to use the tool in their subsequent tasks, as supported by the current findings (Grajcevci & Shala, 2021; Suzuki & Sakurai, 2011). When both operate simultaneously, the self-perceived learning effects and learning strategy effectiveness of motivationally oriented students with high-intensity intrinsic and high-intensity extrinsic goals in mathematics are significantly higher than those of students with low-intensity goals (Lao et al., 2017). In this case, teachers should optimize the design of mathematics courses by combining the characteristics of the technology, integrating the advantages of the technology into mathematics teaching (Attard & Holmes, 2022), encouraging students to maintain curiosity and interest in the mathematical materials provided by GAI, and enhancing students’ mathematics motivation with the help of GAI and other technologies in terms of intrinsic motivation. In addition, mathematics projects, as an important part of the university mathematics curriculum, are also considered by mathematics teachers in terms of how to improve the quality of students’ completion of them with the help of GAI to promote students’ academic achievements in mathematics.

In addition to analyzing the path relationship between the independent and dependent variables, this study also examines the moderating role of mathematics motivation. That is, how the intrinsic psychological characteristics of mathematics motivation would affect the relationships among the variables in the model. The results of this study indicate that strong mathematics motivation would, to some extent, contribute to the needy tendencies of college students in the selection of learning content, the use of tools, and the preference for learning methods. This view is supported by previous research (Kotaman & Aslan, 2020; Maćkowski et al., 2022). When students’ individual demands are met, they are more likely to develop positive usage behaviors, such as active learning and participation in project discussions. Autonomous motivation for mathematics learning influences individuals’ psychological disposition and is directly related to their actual behavior and performance. As confirmed by existing studies (Hagger et al., 2015; Xiao & Sun, 2021), mathematics learning motivation is important for individual behavioral decision-making and implementation processes. Individuals with high levels of mathematics learning motivation are more likely to form positive intentions to use and implement mathematical tools when confronted with mathematical activities.

It can be seen that mathematics motivation plays a key role in college students’ use of GAI to support their mathematics learning process. Mathematics motivation can directly impact the performance of mathematical tasks and potentially influence many other aspects of mathematics learning. Teachers should pay more attention to the value of mathematics motivation, focusing on improving self-awareness and learning strategies by stimulating intrinsic mathematics motivation, strengthening extrinsic mathematics motivation, and combining internal and external motivation to promote college students’ motivation and effectiveness in the process of GAI-assisted mathematics learning to help them to better perform independent mathematics learning and in-depth mathematics learning with the help of GAI.

## 6. Conclusions and Implications

Using the PLS-SEM method, this study aimed to provide a more comprehensive analysis of the factors influencing college students’ use of GAI to support mathematics learning based on the UTAUT model. At the same time, this study also examined the moderating role of mathematics motivation on GAI use. It was found that mathematics motivation, facilitating conditions, individual demand, and behavioral intention positively influenced college students’ GAI usage behavior in mathematics learning. In turn, college students’ behavioral intention to use GAI to support mathematics learning was directly influenced by performance expectancy, personal innovativeness, social influence, and mathematics motivation. Among them, mathematics motivation greatly influenced college students’ behavioral intention and actual behavior of using GAI to support mathematics learning. Meanwhile, mathematics motivation significantly moderated the two path relationships of the structural model: individual demand on usage behavior and behavioral intention on usage behavior.

In the context of the current deep integration of GAI and other emerging technologies in the field of education, this study has significant theoretical value and practical significance in moving beyond the discursive analysis of GAI to the pedagogical application of GAI.

On the one hand, this study constructs a model of the influencing factors to identify college students’ use of GAI in mathematics learning. The explanatory power of up to 70% indicates that the structural model has good analytical and predictive ability and can provide an effective reference for evaluating the influencing factors of college students’ use of GAI. The study systematically analyzes multiple influencing factors of college students’ use of GAI to support mathematics learning through structural equation modeling. This helps to reveal how GAI technology affects the learning process, learners’ cognitive state, and psychological activities. It can not only enrich the theoretical system of the mathematics discipline, pedagogical technology, and the field of AI educational applications but also promote a deeper understanding of the potential of generative AI education.

On the other hand, the results of this study can guide educators to discover the key influences on college students’ use of new technologies in mathematics learning, and help teachers to adjust their teaching strategies. By understanding the factors that influence the use of generative AI to support mathematics learning, students can better identify their shortcomings in using generative AI in the learning process on their mathematics courses, and thus improve their mathematics learning through the effective use of AI tools. For technology developers, understanding the key factors influencing the acceptance and use of GAI technology by key user groups will help them to design AI math tutoring tools that better meet learners’ needs and improve the learning experience.

## 7. Limitations and Future Research

This study also has some shortcomings. First, the current study focuses on college students and analyzes the college student population’s behavioral willingness and actual behavior regarding the use of GAI in mathematics learning. However, GAI is also common in secondary school students’ mathematics learning, and further research could consider extending the methodology of this paper to other educational levels. Second, the survey sample of this study is mainly from the central and eastern regions of China, with fewer samples from the northern and western regions. This may cause the problem of the inapplicability of research results due to regional and school differences, so future research can expand the scope of selecting research subjects and add in-depth interviews or classroom teaching experiment methods. Third, the questionnaire lacks negative descriptions, which limits the attainment of a complete picture of the participants’ views and attitudes.

## Figures and Tables

**Table 1:** Demographic data.

Demographic	Type	Frequency	Percentage
Gender	Male	121	36.56%
	Female	210	63.44%
Age	Freshman	64	19.34%
	Sophomore	51	15.41%
	Junior	106	32.02%
	Senior	83	25.07%
	Postgraduate	27	8.16%
Use GAI in Math Learning	Everyday	17	5.14%
	Frequently	88	26.59%
	Sometimes	176	53.17%
	Seldom	34	10.27%
	Rarely	16	4.83%
Major	Mathematics	220	66.47%
	Engineering	26	7.85%
	Economics	68	20.54%
	Others	17	5.14%

**Table 2:** Descriptive statistics for data normality testing.

Items	Mean	Std. Deviation	Skewness	Kurtosis
	Statistic	Statistic	Statistic	Statistic
PE1	3.73	0.825	−0.384	0.356
PE2	3.70	0.834	−0.338	0.091
PE3	3.69	0.832	−0.323	−0.071
EE1	3.60	0.859	−0.279	−0.131
EE2	3.69	0.807	−0.072	−0.528
EE3	3.57	0.833	−0.185	−0.355
SI1	3.62	0.842	−0.319	0.436
SI2	3.32	0.957	−0.123	−0.346
SI3	3.46	0.944	−0.338	−0.214
FC1	3.80	0.770	−0.479	0.513
FC2	3.73	0.811	−0.260	−0.031
ID1	3.59	0.835	−0.291	−0.160
ID2	3.56	0.852	−0.130	−0.162
ID3	3.66	0.820	−0.462	0.255
ID4	3.58	0.854	−0.290	−0.108
PI1	4.00	0.701	−0.532	0.891
PI2	3.88	0.769	−0.554	0.456
PI3	3.56	0.859	−0.208	−0.041
PI4	3.79	0.828	−0.408	0.050
BI1	3.88	0.738	−0.436	0.916
BI2	3.79	0.768	−0.352	0.327
BI3	3.81	0.786	−0.399	0.480
UB1	3.67	0.785	−0.170	0.035
UB2	3.58	0.760	0.039	0.066
UB3	3.54	0.881	−0.067	−0.449
IGO1	3.73	0.809	−0.530	0.632
IGO2	3.78	0.813	−0.329	−0.146
IGO3	3.78	0.775	−0.348	0.071
IGO4	3.78	0.812	−0.424	0.166
IGO5	3.69	0.769	−0.128	−0.137
EGO1	3.89	0.748	−0.382	−0.003
EGO2	3.71	0.831	−0.346	0.118
EGO3	3.43	0.942	−0.222	−0.227
EGO4	3.45	0.953	−0.310	−0.100
EGO5	3.74	0.816	−0.196	−0.303

**Table 3:** Results of internal consistency, or reliability, and concurrent validity testing.

Constructs	Items	Factor Loading	Cronbach’s Alpha	Composite Reliability	AVE
Performance Expectancy	PE1	0.898	0.874	0.923	0.799
	PE2	0.897			
	PE3	0.887			
Effort Expectancy	EE1	0.877	0.848	0.908	0.767
	EE2	0.862			
	EE3	0.888			
Social Influence	SI1	0.820	0.817	0.891	0.732
	SI2	0.867			
	SI3	0.879			
Facilitating Conditions	FC1	0.905	0.813	0.914	0.842
	FC2	0.930			
Individual Demand	ID1	0.896	0.921	0.944	0.808
	ID2	0.904			
	ID3	0.883			
	ID4	0.913			
Personal Innovativeness	PI1	0.820	0.873	0.914	0.726
	PI2	0.877			
	PI3	0.810			
	PI4	0.898			
Behavioral Intention	BI1	0.911	0.890	0.932	0.820
	BI2	0.879			
	BI3	0.926			
Usage Behavior	UB1	0.895	0.881	0.926	0.807
	UB2	0.908			
	UB3	0.892			
Mathematics Motivation	IGO1	0.808	0.930	0.941	0.613
	IGO2	0.805			
	IGO3	0.773			
	IGO4	0.805			
	IGO5	0.850			
	EGO1	0.752			
	EGO2	0.765			
	EGO3	0.771			
	EGO4	0.702			
	EGO5	0.791			

**Table 4:** Fornell–Larcker criterion values for discriminant validity testing.

	PI	ID	UB	FC	EE	MM	SI	PE	BI
PI	0.852								
ID	0.667	0.899							
UB	0.702	0.716	0.899						
FC	0.655	0.583	0.751	0.917					
EE	0.705	0.781	0.724	0.652	0.876				
MM	0.738	0.726	0.781	0.684	0.745	0.783			
SI	0.552	0.606	0.656	0.583	0.589	0.670	0.856		
PE	0.626	0.712	0.666	0.579	0.729	0.655	0.517	0.894	
BI	0.722	0.635	0.765	0.743	0.678	0.778	0.613	0.653	0.905

**Table 5:** Heterotrait–Monotrait Ratio for additional discriminant validity testing.

	PI	ID	UB	FC	EE	MM	SI	PE	BI
PI									
ID	0.745								
UB	0.802	0.795							
FC	0.776	0.672	0.882						
EE	0.820	0.886	0.835	0.780					
MM	0.813	0.787	0.861	0.778	0.839				
SI	0.651	0.697	0.773	0.714	0.708	0.771			
PE	0.714	0.791	0.757	0.685	0.846	0.722	0.609		
BI	0.818	0.700	0.863	0.875	0.777	0.844	0.715	0.739	

**Table 6:** Hypothesis testing and path coefficient.

Hypothesis		Path Coefficient (*β*)	Sample Mean	Standard Deviation	T Statistic	*p* Value	Interpretation (*p* < 0.05)
H1	Performance Expectancy→Behavioral Intention	0.178	0.180	0.063	2.813	0.005	Sig.
H2	Effort Expectancy→Behavioral Intention	0.046	0.046	0.081	0.567	0.571	Not Sig.
H3	Social Influence→Behavioral Intention	0.120	0.117	0.059	2.042	0.041	Sig.
H4	Facilitating Conditions→Usage Behavior	0.290	0.290	0.057	5.117	0.000	Sig.
H5	Personal Innovativeness→Behavioral Intention	0.257	0.250	0.067	3.815	0.000	Sig.
H6	Individual Demand→Behavioral Intention	−0.067	−0.069	0.077	0.873	0.383	Not Sig.
H7	Individual Demand→Usage Behavior	0.232	0.234	0.063	3.703	0.000	Sig.
H8	Behavioral Intention→Usage Behavior	0.204	0.193	0.081	2.527	0.012	Sig.
H9	Mathematics Motivation→Behavioral Intention	0.406	0.413	0.087	4.672	0.000	Sig.
H10	Mathematics Motivation→Usage Behavior	0.255	0.264	0.078	3.276	0.001	Sig.

**Table 7:** Direct, indirect, and total effects in the model.

Factor	Determinant	Effect		
		Direct	Indirect	Total
Behavioral Intention (BI) (R^2^ = 0.680)	Performance Expectancy	0.178	0	0.178
	Effort Expectancy	0.046	0	0.046
	Social Influence	0.120	0	0.120
	Individual Demand	−0.067	0	−0.067
	Personal Innovativeness	0.257	0	0.257
	Mathematics Motivation	0.406	0	0.406
Usage Behavior (UB) (R^2^ = 0.739)	Personal Innovativeness	0	0.052	0.052
	Individual Demand	0.218	0	0.218
	Facilitating Conditions	0.290	0	0.290
	Effort Expectancy	0	0.009	0.009
	Mathematics Motivation	0.338	0	0.338
	Social Influence	0	0.024	0.024
	Performance Expectancy	0	0.036	0.036
	Behavioral Intention	0.204	0	0.204

**Table 8:** Results of moderating effect testing.

Regression Analysis (n = 331)		Standardized Regression Coefficient (*β*)	T Statistic	*p* Statistic
Dependent Variable	Independent Variable			
Usage Behavior				
	Gender	−0.033	−1.007	0.315
	Age	0.024	0.715	0.475
	ID	0.317	6.617 ***	0.000
	MM	0.525	10.819 ***	0.000
	ID × MM	0.079	2.305 *	0.022
Usage Behavior				
	Gender	−0.050	−1.588	0.113
	Age	−0.018	−0.562	0.575
	BI	0.430	8.861 ***	0.000
	MM	0.435	8.846 ***	0.000
	BI × MM	0.080	2.436 *	0.015

Note: * *p* < 0.05, *** *p* < 0.001.

## Data Availability

Data are available from the corresponding author on reasonable request.

## Abbreviations

The following abbreviations are used in this manuscript:

GAI	generative artificial intelligence
UTAUT	the Unified Theory of Acceptance and Use of Technology
PLS-SEM	partial least squares structural equation modeling

## Appendix A

**Table A1:** Questionnaire constructs and items to analyze intentions to use and actual use of GAI in mathematics learning.

Items	Constructs and Contents	Reference
	Performance Expectancy (PE)	(Venkatesh et al., 2012; Wijaya et al., 2022c)
PE1	I think GAI is very helpful in my math studies.	
PE2	GAI can increase my chances of gaining important knowledge in math learning.	
PE3	GAI can effectively inspire me to solve math problems and give me problem-solving ideas.	
	Effort Expectancy (EE)	(Venkatesh et al., 2012; Zeng, 2019)
EE1	I find it easy to use GAI in my math studies.	
EE2	GAI provides math information that is easy to understand.	
EE3	I feel that the GAI platform has good interactive performance in math learning.	
	Social Influence (SI)	(Venkatesh et al., 2003, 2012)
SI1	My teacher encouraged me to use GAI to help me learn math.	
SI2	I use GAI because most of the students in my class use it.	
SI3	I use GAI because mainstream media recommend it.	
	Facilitating Conditions (FC)	(Venkatesh et al., 2003, 2012)
FC1	I have the necessary electronic equipment and network environment to use GAI for learning mathematics.	
FC2	I have the basic knowledge to use GAI for math.	
	Individual Demand (ID)	(Gao, 2023)
ID1	GAI can identify the math materials I need based on the problem.	
ID2	GAI can recommend highly adapted math problems based on my learning situation.	
ID3	GAI can gradually understand my math learning needs over successive sessions.	
ID4	GAI plays the role of a good teacher and peer in solving my math problems.	
	Personal Innovativeness (PI)	(Tian et al., 2024; Wijaya et al., 2022b)
PI1	I am willing to use new technology in my math learning.	
PI2	When I hear about a new technology, I am curious to experience it and feel how it can help my math learning.	
PI3	I usually try new technologies for learning mathematics earlier than my peers around me.	
PI4	When a new learning technology is available, I will use it to learn mathematics.	
	Behavioral Intention (BI)	(Alzahrani et al., 2019)
BI1	I am willing to continue using GAI in my math learning if the content is appropriate.	
BI2	I would be willing to try using GAI in the learning process of other courses.	
BI3	I would recommend that a friend use GAI for math learning.	
	Usage Behavior (UB)	(Wijaya et al., 2022c; Yuan et al., 2023)
UB1	I am relatively satisfied with the effectiveness of my use of GAI.	
UB2	I have extensive experience using GAI technology for learning mathematics.	
UB3	Using GAI has become part of my daily math learning routine.	

**Table A2:** Mathematics motivation scale of applying GAI for learning among college students.

Items	Constructs and Contents	Reference
	Intrinsic Goal Orientation (IGO)	(Pintrich, 1991; Zheng et al., 2024)
IGO1	I tend to choose more challenging math problems and materials provided by GAI.	
IGO2	I want GAI to provide math course materials that stimulate curiosity, even if they are not easy to learn.	
IGO3	The most satisfying thing for me is the help GAI can provide in promoting a thorough understanding of what I am learning.	
IGO4	I wish GAI had more extensive homework and project materials, even if they don’t necessarily improve my math grades immediately.	
IGO5	Most of the time, I get satisfaction and a sense of accomplishment from GAI’s help learning math.	
	Extrinsic Goal Orientation (EGO)	(Liu & Lin, 2010; Pintrich, 1991)
EGO1	I hope using GAI will help me improve my academic performance in math.	
EGO2	I hope using GAI will help me achieve higher grades in my math courses than most classmates.	
EGO3	When learning math, I use GAI to show others how well I can learn math.	
EGO4	I expect to receive praise from my teacher or recognition from my classmates for using GAI in my math learning.	
EGO5	Using GAI in math will help me get into the college I want to attend.	
